# The Immune System Computes the State of the Body: Crowd Wisdom, Machine Learning, and Immune Cell Reference Repertoires Help Manage Inflammation

**DOI:** 10.3389/fimmu.2019.00010

**Published:** 2019-01-22

**Authors:** Irun R. Cohen, Sol Efroni

**Affiliations:** ^1^Department of Immunology, Weizmann Institute of Science, Rehovot, Israel; ^2^Faculty of Life Sciences, Bar-Ilan University, Ramat-Gan, Israel

**Keywords:** immune computation, swarm intelligence, machine learning, autoreactive repertoires, T cells, autoantibodies

## Abstract

Here, we outline an overview of the mammalian immune system that updates and extends the classical clonal selection paradigm. Rather than focusing on strict self-not-self discrimination, we propose that the system orchestrates variable inflammatory responses that maintain the body and its symbiosis with the microbiome while eliminating the threat from pathogenic infectious agents and from tumors. The paper makes four points:

1. The immune system classifies healthy and pathologic states of the body—including both self and foreign elements—by deploying individual lymphocytes as cellular computing machines; immune cells transform input signals from the body into an output of specific immune reactions.
2. Rather than independent clonal responses, groups of individually activated immune-system cells co-react in lymphoid organs to make collective decisions through a type of self-organizing swarm intelligence or crowd wisdom.
3. Collective choices by swarms of immune cells, like those of schools of fish, are modified by relatively small numbers of individual regulators responding to shifting conditions—such collective inflammatory responses are dynamically responsive.
4. Self-reactive autoantibody and T-cell receptor (TCR) repertoires shared by healthy individuals function in a biological version of experience-based supervised machine learning. Immune system decisions are primed by formative experience with training sets of self-antigens encountered during lymphocyte development; these initially trained T cell and B cell repertoires form a Wellness Profile that then guides immune responses to test sets of antigens encountered later. This experience-based machine learning strategy is analogous to that deployed by supervised machine-learning algorithms.

We propose experiments to test these ideas. This overview of the immune system bears clinical implications for monitoring wellness and for treating autoimmune disease, cancer, and allograft reactions.

## The Immune System Manages Inflammation

In the beginning, it was taught that the function of the immune system was to distinguish between the self and the foreign—whatever was foreign was to be rejected and, in contrast, what belonged to the self was to be ignored (1). We need not bother to define the tricky terms *self* and *foreign* (2) because we now know that the functions of the immune system are much more varied than a simple self-not-self binary distinction (3, 4): The immune system clearly protects the body from invading pathogens, but it also welcomes and manages our symbiosis with the essential bacterial microbiome and viral components of the body (5); the immune system also heals wounds and repairs injuries to maintain us in the face of the accidents of life (6, 7); it detects and destroys aged cells and transformed tumor cells (8, 9); and it rejects tissues transplanted from allogeneic individuals, while tolerating our foreign symbionts (10).

These complex functions of the immune system can be reduced to a common process: in one way or another, all the effects of immune activity involve the management of what is called *inflammation* (6). Where grossly visible, inflammation is marked by redness and swelling due to changes in tissue blood flow and edema; microscopically, inflammation is marked by accumulations of immune system cells; by the death and growth of many types of cells; by the proliferation of scar-forming connective tissues; and, often, by the regeneration of blood vessels and damaged tissue cells. The process of inflammation usually terminates when the injury heals, but sometimes an inflammatory process persists chronically or periodically exacerbates, or may develop unnecessarily in otherwise healthy tissue. In these instances, the inflammatory process itself can be the cause of disease—autoimmune diseases result from such misguided inflammatory processes.

## The Immune System Classifies the State of the Body

In the beginning, it was thought that an adaptive immune response was the exclusive property of individual antigen-specific lymphocyte clones, each bearing an antigen receptor of a single specificity (11). The population of mature lymphocytes was presumed to be purged during development of receptors that could possibly recognize molecules of the host (self-antigens); mature lymphocytes could recognize only foreign antigens. But, as we mentioned above, body maintenance obliges the immune system to interact with self-molecules as well as with not-self- molecules. Immunity is not merely a reflex to a foreign presence, but an act of cognition (12).

**Figure 1:**
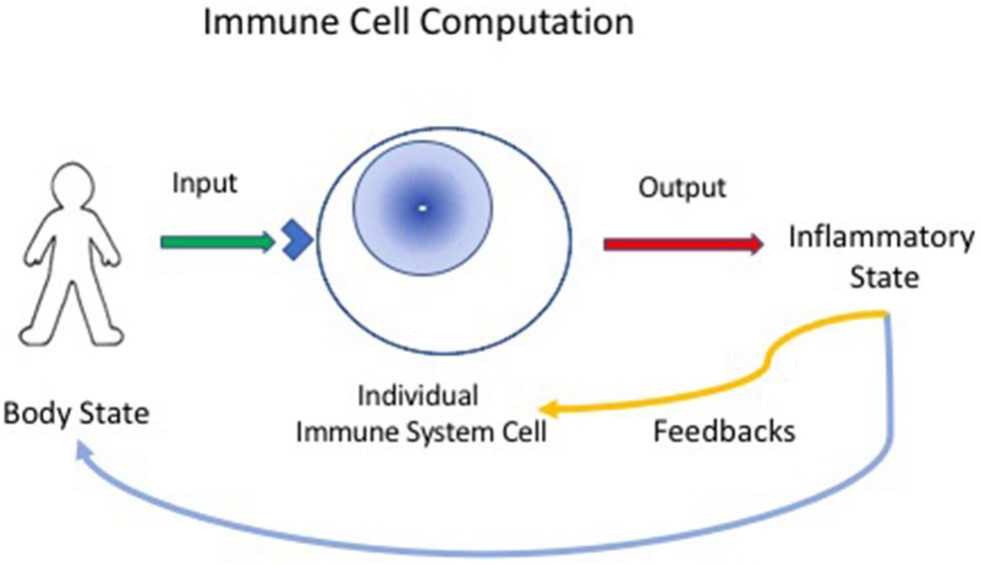
Immune cells compute the state of the body. Individual immune cells bear receptors (blue corner on cell surface), innate receptors or antigen receptors, which are activated by input signals from the body. The individual responding cell, innate, or adaptive, then transforms the input signals into outputs that can mediate inflammatory responses. The inflammatory state then feeds back to heal or protect the body. The immune response also provides feedback to immune system cells and tissues—thus, the immune system organizes itself through experience.

Now, if we define computation as the ordered transformation of input into output (13), we can perceive the immune system to be a computational, living reactive system (4, 14); the system gathers input about the state of the body, locally, and generally, and reacts to arrange an output of appropriate inflammatory procedures that feedback on the body to maintain, heal, regenerate and protect it; immune experience also feeds back to modify the immune system itself (Figure 1).

Immune computation differs in many ways from computer-based algorithms and classifiers: First, note that *the hardware is the software*; the programed activities of the molecules, cells and organs comprising the physical system actually constitute functioning algorithms. The *performer* and the *program* are identical—a living cell is defined by the way the cell's components behave programmatically.

Secondly, computation is *distributed* throughout a living body*;* each immune system cell computes in parallel; each cell (lymphocyte, macrophage, dendritic cell, stem cell, endothelial cell, etc.) receives whatever signals its array of receptors can detect; each cell then responds to transform (compute) its input information into an output of signal molecules, receptors, metabolic reactants, antibodies, or other products that comprise an inflammatory output (Figure 1). The response of the cell and its outputs are determined by the state of the individual computing cell; this state reflects the cell's differentiation and its history, along with the input to the cell from other cells and molecules. In other words, immune system cells have no central processor—each cell is its own information processor.

The clonal selection paradigm focuses on the behaviors of individual, receptor-bearing lymphocytes, and clones. Individual cells, however, must integrate their disparate behaviors to generate a systemic decision; an ordered immune response emerges from the way a collective of cells integrate their behaviors—a type of *swarm intelligence* or *crowd wisdom* (15). Immune crowd wisdom emerges from crowds of cells, including T cells and B cells that bear each its own antigen receptor along with other types of immune system cells that express only innate receptors and do not recognize antigens at all. Moreover, collectives of responding cells have to dynamically adjust their system-wide behaviors as the inflammatory situation changes over time for better or worse. How do immune crowd behaviors take place?

## Co-respondence, Bystander Cells, and Immune Anatomy

Each immune system cell is exposed to only a partial and limited view of its surroundings—the cell's perceptions are dictated by the particular receptors expressed by the cell and the ligands impinging on them. Even a specific antigen receptor can tell only a partial story: any antigen receptor can see only an epitope fragment or domain of the antigen that may or may not have originated from an infection, a tumor, an injury, or a healthy tissue. Moreover, a single T-cell receptor has been estimated to be able to interact functionally with many different peptides with varying avidity (3); how then can a T cell know which of its potential antigen epitopes it is seeing? Innate receptors borne by lymphocytes and other immune cells are also restricted to particular domains of their ligand molecules. A lone cell, necessarily, is blind to information that does not activate its receptors—each cell is confined to a world compressed by its own shortsightedness.

Moreover, just as a single clone has a limited view of the world, a single clone is not sufficient to effect an immune response; an appropriate inflammatory response requires the participation of large collectives of a variety of different cells. The doubling time of a T cell is about 10 h; a single T cell simply cannot generate enough progeny in the time needed to respond to an infection or potential tumor. How are individually limited views integrated to generate a diagnostic consensus and how can a coherent and dynamic multi-cellular inflammatory response be mobilized in a relatively short time?

### Co-respondence

**Figure 2:**
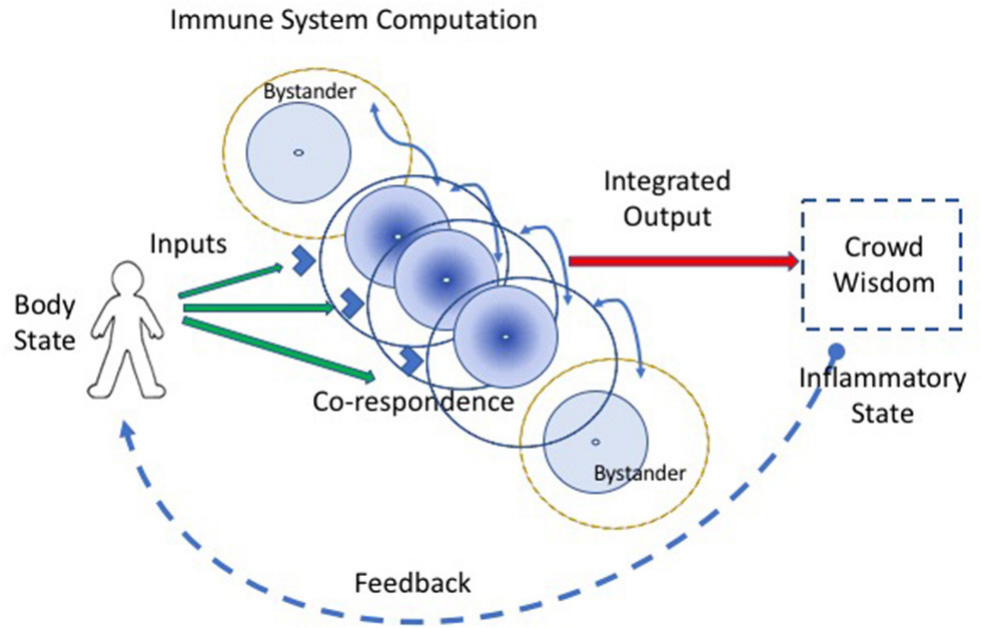
Co-respondence between cells generates immune computation at the systemic scale. An integrated immune response results from the ability of individual immune cells to tune their inflammatory responses in the light of the responses they sense from adjacent cells (the curved blue lines connecting cells); this co-respondence is mediated by innate receptors. Bystander cells, which may lack direct input signals from the body, are enlisted into the response by co-respondence with adjacent immune cells.

*Co-respondence* helps (Figure 2). Co-respondence describes the ability of lone immune cells to sense and respond to the states of adjacent immune and body cells (3); this mutual responsiveness generates a type of *swarm intelligence* or *crowd wisdom*. By interacting with neighboring cells, a collective of immune cells together can construct a relatively broad assessment of the situation. A cell may not see the antigens or other signals perceived by adjacent immune cells, but each cell can sense, by its receptors for cytokines, metabolic products, and other innate response mediators, the state and degree of activation of adjacent cells. The collective of cells, one-by-one, is able to modify its local behavior according to the output signals of the collective crowd wisdom. An integrated crowd response arises from the mutual summation of adjacent responses (16). The input string of individual antigens and mediator molecules is thus transformed into a collective computation.

**Figure 3:**
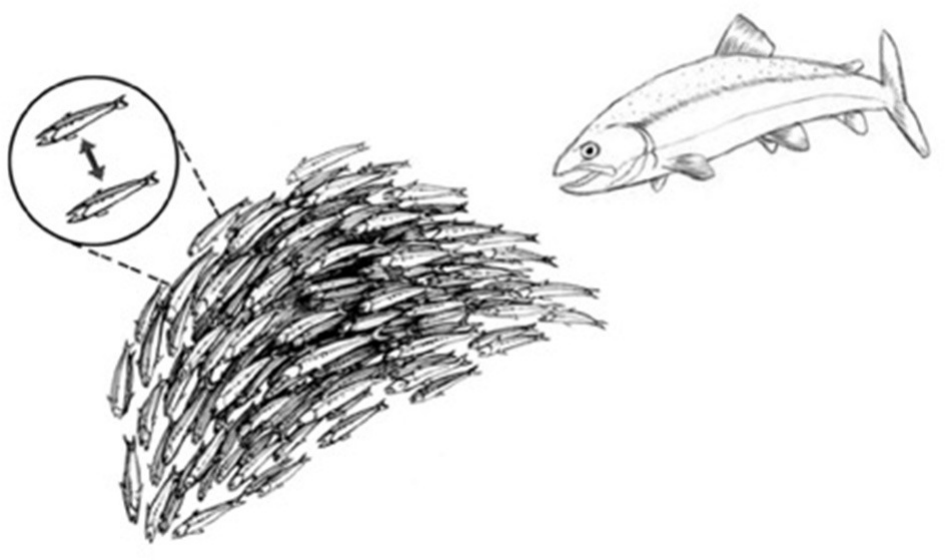
Schools of fish manifest self-organized behavior. A school of fish, as an organized group, effectively flees from a predator fish. The dashed-line inset shows that the shape and direction of the school of fish is actually self-organized by a relatively small number of visual signals exchanged between adjacent fish.

**Figure 4:**
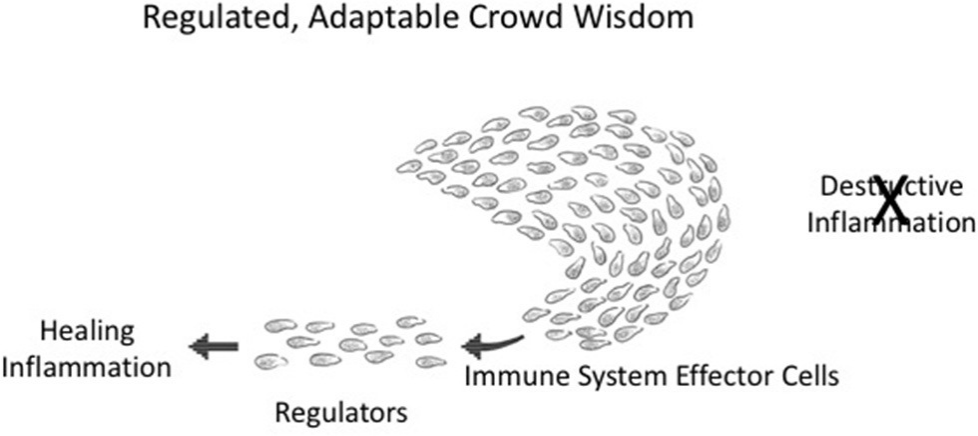
Adaptable crowd wisdom. A swarm of immune cells is depicted as a school of fish initially proceeding in the direction of *Destructive Inflammation*; but a small number of *Regulators* (by spreading Co-respondence; see Figure 3) can shift the behavior of the entire swarm toward Healing Inflammation.

This strategy for achieving system-wide integration of piecemeal perceptions is common throughout nature. Schools of fish, colonies of ants, migrating locusts, and flocks of birds (and even relatively simple robots) can exhibit collective responses that appear to be miraculously coordinated and highly complex (Figure 3). Yet upon examination and mathematical modeling, these collective behaviors turn out to be the products of relatively simple cues transmitted between adjacent individuals (16). Such collective behaviors do not require an external, all-knowing manager to impose its will on the group; the collective of individuals *self-organizes* (17). A mutually interacting collective of individuals may appear to define a goal, as it were, and can manifest complex, seemingly goal-directed behavior merely by the exchange of relatively simple signals between adjacent individuals (Figure 3, dashed line inset). Local signaling then spreads through the group as a kind of integrating epidemic (from the Greek *epi*—upon; *demos*—the population). Biological self-organization emerges, as it were, from crowd wisdom. The epidemic spread of local cell responses, like the spread of information in a school of fish or flock of birds, quickly leads to highly coordinated group “decisions” that effectively integrate the individual immune cell responses into a collective inflammatory response—a few initiating immune cells mobilize bystander, crowd support (Figure 4).

Integrated collective immune responses need to finetune themselves as the environment changes—greater or lesser tissue damage, many or few infectious agents, the evolving state of a tumor, the mending of a broken bone, and so forth. This integrated *crowd behavior* can be adjusted on the run by a few regulator cells in the collective who have sensed a change in the infection or in tissue healing; adjacent neighbors adjust their responses, which then spread to the other participants in the immune response. The immune system, like a school of fish or a crowd of people, is dynamically adaptive. Figure 4 depicts an about-face shift in collective direction from *Destructive Inflammation* to *Healing Inflammation*, brought about by a small number of regulatory individuals who have sensed the need for change. Such manipulations of group inflammatory behavior by small numbers of regulatory elements is termed “infectious tolerance” (18); indeed, a few percent of Tregs are all that is needed to influence major inflammatory decisions (19).

### Bystanders

*Bystander activation* refers to the fact that most of the activated lymphocytes and other leukocytes accumulating at an inflammatory site do not bear antigen- receptors specific for antigens borne by the agent that triggered the inflammation (20). Unfortunately, the word *bystander* bears a negative connotation—the cells that migrate to the site of the antigen without receptors for the antigen, in the eyes of the classical clonal selection theory, don't belong there. They are merely chance lookers on. But we now know that co-respondence is of the essence—bystanders are the expression of crowd wisdom; it's the way the immune system works. The informed few who see the antigens arouse a cohort of “bystander” cells to help mediate the inflammation (Figure 2). Crowd wisdom is an integral part of immune computation of body state.

### Immune Anatomy

The functional anatomy of the immune system is a key factor in integration and decision making. The immune system in real life, unlike our laboratory experiments, is not a culture of cells dispersed in a flask—the immune system is organized anatomically into defined organs (lymph nodes, bone marrow, thymus, spleen, Peyer's patches, etc.), which are connected by specific flows of molecules and cells in blood vessels, lymph vessels, and extracellular fluids (3). Cells and molecules do not meet merely by chance; immune interactions are organized in space and time by anatomic structure, flow, and signaled migrations—organized interactions are analogous to “hard wired” connections. Thus, collective decision-making and immune response phenotypes are decisively organized by the anatomical infrastructure of the system—machine learning, as we shall discuss below, emerges from this organization. The anatomic details are beyond the scope of this bird's-eye overview. Here, we only direct attention to the importance of “anatomically wired” influences on immune decision making.

## Immune Machine Learning

Mainstream immunology, steeped in the clonal selection theory of adaptive immunity (21), has tended to attribute regulation of the immune response to single clones of lymphocytes and their antigen receptors; binding a specific antigen triggers a response—no antigen or antigen receptor, no response (Figure 1). Our present discussion of immunological swarm intelligence and crowd wisdom (Figures 2–4) connects immune system behavior by analogy to the collective behavior of schools of fish, flocks of birds, and hives of bees along with other collective biologic entities. What is the basis of this immune group behavior? Note that the immune system is uniquely like the brain; both brain and immune system develop fully, far beyond their genes, as a result of somatic lifetime experience (3). In this section, we would like to suggest that immune experience requires preliminary training reminiscent of *supervised machine learning*.

What is machine learning? The term machine learning was coined to describe the way an algorithm running on a computer can be used to uncover meaningful patterns hidden in diverse sets of data. Supervised Machine learning is a type of pattern recognition in which previous training subsequently enables detection of informative patterns buried in test sets of new data (22). The computer algorithm is first educated by way of primary interactions with selected training sets of model data. The machine learns to identify correlations or statistical associations between the component entities that comprise the data included in its training sets.

Unlike a computer algorithm, the immune system does not process electronic signals: Antigens, metabolic products, cell interaction molecules, and other molecular signals make up the sets of data perceived by the cells of the immune system. The correlations between the components comprising a set of data can be very subtle and obscure to the human observer, yet such correlations are detectable by machine learning algorithms, and, by analogy, by networks of cells and antibodies in the immune system. As a consequence of exposure to training sets of input, the computer algorithm—and the immune system—can accumulate a bank of learned correlations. These formative correlations can then be used by the computer or the repertoire networks of the immune system to interpret new test data.

### Learning Similarities

Interpretation of new data emerges from the presence or absence in the data of correlations previously learned during primary training. A preexisting algorithm is not needed to learn each individual pattern of components; the machine or the biologic system need only be programmed generally to detect any patterns shared by both the learning and test sets of data. A characteristic feature of one type of machine learning—*deep neural networks*–is the interaction between multiple sets of hidden networks that process the input. The current science of deep learning does not completely understand how such network architectures actually work to interpret patterns of input, and we cannot get into the arcane details here. The important point is that it works.

**Figure 5:**
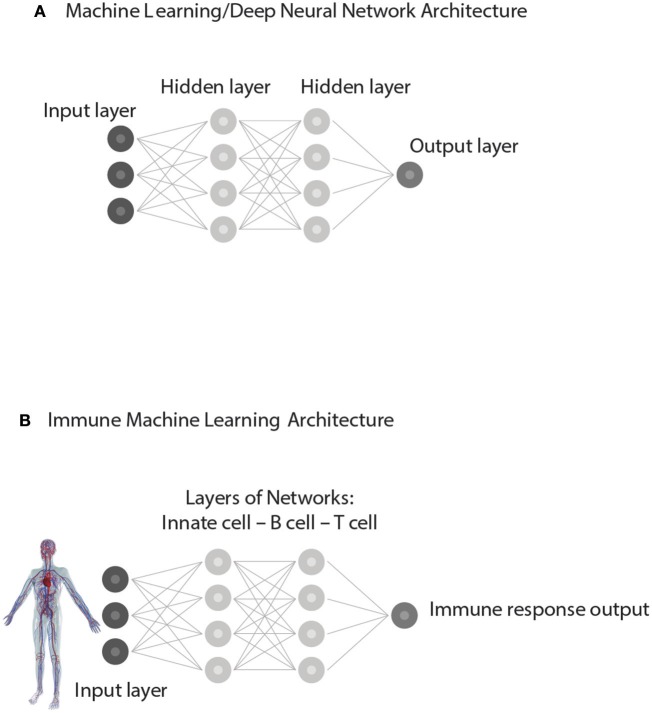
Supervised Machine learning from experience. **(A)** represents a simplified Architecture of computer Machine Learning in which Hidden Layers of interacting networks are organized as Deep Neural Networks. Input information is interpreted by comparing the present test Input to training sets of information previously experienced. The processing of test Input through hidden layers of networks generates an informative Output. **(B)** represents an analogous Immune supervised Machine Learning Architecture. Input from the body—healthy or ill—is gathered by receptors of immune cells and processed through interactions organized as hidden layers of networks of Innate cells, B cells, and T cells. By comparing the input data to the training data obtained during immune cell development, the immune system generates an immune response suitable to the situation. The output feeds back to serve the needs of the body and to update the internal organization of the immune networks themselves.

The new data may appear to the human observer to be new, but the correlations, through prior training, are already familiar to the computer or to the acquired repertoires of the immune system; in a word, the new data are not new to the expert system—artificial or biological. Similar patterns in the training and test sets of input data are uncovered by a process involving iterations within and between different levels of hidden, internal networks organized within the deep neural network (Figure 5). In other words, the immune responses to test sets of antigens are *supervised*, as it were, by the training sets of immune activation experienced during development.

The power of artificial deep neural networks to deal with complexity is evident in image analysis and in natural language processing. The ability of driverless cars to negotiate their way through traffic requires precise, dynamic image analysis; refinements are still needed, but the technology promises to significantly change human transportation. Similarly, the ability of computers to process natural language will significantly influence human culture. Likewise, smart houses will use deep neural networks to affect the way we live. As we mentioned above, experts are still not sure how deep neural networks work and how they succeed where other methods have failed. Some have gone as far as calling machine learning “alchemy” or “alien technology” (23). We know how to build and use them, but we do not know exactly how they do what they do.

Deep learning “black boxes” are now built using about 150 million parameters. This is a large parameter space, and it may explain why such machine learning models have outgrown our ability to understand precisely how they work. Note, however, that networks comprising 150 million parameters express only a fragment of the complexity available for computational use by the immune system. For example, a milliliter of blood contains 2 million T cells; each T cell expresses tens of thousands of proteins on its surface. Add to that the additional dimension of spatial changes over time, and even a droplet of blood contains orders of magnitude more complexity than one of the larger deep learning networks, such as the VGG19 model (24).

Don't let the term *machine learning* mislead you: living systems do not use computer algorithms and are not machines in the way that computers are machines (artificial computers made of DNA are in very early stages of development). Fortunately, your brain serves as a familiar example of a biological learning machine. Consider the fact that you are able to recognize a familiar three-dimensional face when you see it as a two-dimensional cartoon because layers of networks deep in your brain are able to detect a similar pattern of key face features shared by both the real face and the caricature. You can use a map to drive your car through a new environment because your brain has learned to see common patterns shared by the map and the real world perceived by your eye—a map is a caricature of a landscape. Past experience has taught your brain to extract essence from accident. Likewise, Google Photos uses machine learning algorithms to recognize and catalog the photographed faces of an individual as he or she proceeds from childhood into old age; the person is identifiable both by computer algorithm and our brains despite the marked changes in physiognomy during aging. (Indeed, the Google algorithm can help reveal relationships hidden in brains: one of us finds it most intriguing that Google clustered photos of a daughter-in-law with photos of one's daughters—was a son's spouse preference trained by early visual input training from his sister or his mother?).

### Learning Differences

Conversely, prior experience with learning sets of data can also teach your brain to detect meaningful *differences* between grossly similar signals. For example, the more familiar you are with a set of monozygotic “identical” twins, the easier it is for you to tell them apart, even when they are not both present for side-to-side comparison. Indeed, very subtle differences are often easiest to detect on a background of close similarity—a minor difference in the strips or stars of army rank is most visible when all the soldiers wear grossly similar uniforms. Amotz Zahavi has claimed that the vividly colored markings on bird species evolved to enable females to see genetic differences between apparently similar male suiters (25). We here propose that early training enables the immune system, like the brain, to detect meaningful differences as well as similarities.

The ability of your immune system to distinguish, for example, a symbiotic bacterium from a pathogenic bacterium requires the recognition and distinction of particular input patterns present in the myriads of molecular signals impinging on your collectives of immune cells. Both pathogenic bacteria and bacteria of the symbiotic microbiome express LPS or peptidoglycans and both types of bacteria share a great many other foreign antigens and innate signals; but the invading pathogen damages the host and so appears accompanied by signals produced by damaged body tissues and by metabolic changes (5). By profiling the mixture of bacterial and body signals, your immune system can discriminate between very similar bacteria by attending to informative differences in patterns of signals—a lone antigenic signal rarely suffices for a definitive diagnosis.

Your immune system can also sense patterns of antigens compatible with general health; markedly different tissues like lungs, hearts and kidneys can signal a pattern of health, despite their obvious differences in molecular structure and behavior. Just as there is a diagnostic profile difference between infectious pathogens compared to similar symbionts, there is a profile of similarity that designates health in highly dissimilar body organs. Indeed, we have recently learned that growing tumors may trick the immune system into tolerating them as normal tissue despite their abnormal mutations—the tumors express health signals that prevail over tumor signals and neoantigens that might otherwise expose a state of pathology; the tumor, as it were, exploits profiled signals of well-being that enable it to masquerade as healthy tissue (26). Fortunately, the tumors in some individuals, in due course, can become targets for spontaneous immune destruction, or medically engineered destruction in response to anti-checkpoint immunotherapy (27).

### Two Requirements for Immune System Supervised Machine Learning

In summary, deep learning requires two elements: data for training and networks for data processing. *Training sets* of data provide the immune system with reference criteria for interpreting new data; processing the data emerges from layers of *network interactions* that take place deep within the system. Experimental evidence shows that healthy individuals share autoreactive TCR and autoantibody repertoires. The clonal selection paradigm cannot explain the possible function of this healthy, immune self-reactivity; here we propose that these repertoires serve to supervise a type of immune machine learning.

### Training Sets

Immune supervised machine learning requires training sets of antigen experience that initially prime the immune system for its subsequent performance in dealing with test immune challenges that arise when confronting the real world. The initial T cell and B cell training repertoires both arise early during development in isolated body locations protected from the environment; this adaptive learning is driven by healthy self-antigens.

The primal TCR repertoire develops in the thymus through genetically programed experience with self-antigens expressed, processed and presented by innate dendritic cells (28). Thymic T-cell development has been studied in detail for some decades and much is known about it (29). There is no need to recount the details here; the bottom line is that programed thymic selection to particular self-antigens is critical to the normal development of the mammalian immune system (30); faulty thymic T-cell development can lead to autoimmune disease and immune system deficiency in dealing with pathogens (31). T-cell experience with a healthy self-training set of antigens is necessary (but, alas, not sufficient) for developing a healthy immune system. The specificity of healthy self-antigen training is exemplified by mutations in AIRE and other transcription factor genes that lead to severe autoimmune disease resulting from the lack of expression of certain tissue antigens by thymic epithelial cells (32). Note that T-cell development in the thymus is associated with TCR repertoires that are shared by different individual humans; some of these *public* TCR structures are identical in humans and mice and are organized in networks of very similar amino acid sequences (33).

The primal B cell repertoire has been much less studied than has the primal T-cell repertoire. Early studies of autoantibodies in the bloods of healthy subjects were done using relatively crude western blot technology (34). Most relevant to immune system computation are recent antigen-microarray studies of autoantibody repertoires in the bloods of young mothers and in the cord bloods of their healthy newborns. The antibodies in cord blood are important because they reflect initial training of the B-cell repertoire with which the newborn faces life outside the safety of mother's womb. We have carried out two such studies: the first used 10 mother-cord pairs (35) and the second used 71 mothers and their 104 newborns; we measured IgG and IgM antibody binding to 295 self-antigens, compared to 27 standard foreign antigens (36). The results have been published; here we briefly summarize the key findings:

1. The binding of some cord blood autoantibodies to self-antigens is at least as strong as the binding of maternal antibodies to some foreign antigens; thus, the congenital autoantibody repertoire recorded by microarray technology appears to reflect significant immune priming to healthy self-antigens.
2. Because maternal IgG is actively transported across the placenta to the developing fetus, the IgG repertoire of each newborn is strongly correlated with that of its mother; there is relatively less correlation between the IgG repertoires of different newborns or different mothers.
3. Human newborns manifest a strong correlation of IgM autoantibody repertoires amongst themselves as a group that differ from the IgM repertoires of each of their mothers. In contrast to maternal IgG antibodies, antibodies of the IgM isotype do not cross the placenta from mother to fetus (37). Hence, any IgM autoantibodies in cord blood had to have been produced by the fetus during development in the isolation of the womb. Thus, genetically diverse human babies undergo B-cell training experience to develop standard repertoires of IgM autoantibodies during pre-natal life. Healthy autoantibody repertoires, like public T-cell repertoires, manifest networks (38) of connectivity linking certain dominant self-antigens (33).

At the present time, we do not know of early training experiences of innate leukocytes, which do not bear receptors for antigens. However, dendritic cells, epithelial cells and probably other innate cells do participate in the training of the adaptive T-cell and B-cell repertoires (39)—it remains to be seen if this early innate-cell experience also trains innate leukocyte development.

### Layers of Network Interactions

The second element essential to machine learning algorithms based on neural networks is an architecture that features multiple layers of interacting networks that process input data (Figure 5A). In computer parlance these deep layers of interacting networks have been termed “hidden”; the internal networks in living systems such as the brain and the immune system are molecular and they too are essentially “hidden” from view. Figure 5B depicts network interactions between innate cells, T cells and B cells *as if* they were deep layers of immune processing. Advanced imaging technics can show the movements and contacts of groups of individual cells, but we have no way, yet, of observing the information transferred between such interacting cells nor can we see the molecules involved. Experiments teach us that innate antigen-presenting cells interact with T cells and B cells, and that T cells and B cells interact between themselves in various ways. Moreover, T cells of various types interact with other T cells and B cells and antibodies interact with each other (33, 40); through regulatory (41), idiotypic (42), ergotypic (43), and other types of network connections. The anatomy of lymphoid organs includes discreet layers of interacting cell types, as we mentioned briefly above. Here, we propose that this architecture of anatomically layered immune networks has evolved to materialize a biologic version of experience-based machine learning.

Classically, the existence of networks of interacting cells and molecules has been explained *ad hoc* by the need to satisfy a list of functional binary distinctions in the immune response: IgM vs. IgG antibodies; innate vs. adaptive recognition; memory vs. transience; helpers vs. killers; suppressors vs. effectors; Th1 vs. Th2 helper types; and so on and so forth. Each newly discovered cell or interaction was assigned to fulfill a singular need, a particular goal, to account for its evolution. Immunology had no single organizing principle, or fundamental strategy that would make sense of all the system's seemingly redundant complexity.

Here, we support the idea that these sets of interacting immune elements serve immune decision-making by constituting a multi-level network architecture that serves experience-based supervised machine learning. Like a deep learning machine learning contrivance, the immune system is organized to include multiple levels of interacting cells and molecules triggered into motion by an immunological experience, which is them interpreted by reference to early training sets of data. Obviously, other explanations are conceivable; experimentation is needed.

## Machine Learning and Immune Wellness

Note that the primary immune reference repertoires selected during early development of B-cell and T-cell repertoires arise through interactions with healthy tissues; we can reason that the emerging repertoires of selected lymphocytes signify a pattern of health—it is reasonable to hypothesize that healthy self-antigens are what lymphocytes see in the thymus and *in utero*. In other words, the adaptive immune system is first trained to recognize relative wellness. Consequently, the similarity of a profile of test antigens to the training set profile means that all looks well and no destructive inflammation is needed. In contrast, a functional dissimilarity of a test antigen pattern to the healthy reference pattern should spur the immune system into inflammatory action (Figure 5B). Hypotheses do their job by inviting experimentation, and the existence of a positive wellness profile needs experimental support. Below, we shall suggest some novel experiments and predictions.

We can view the immune supervised machine learning process as a *wellness theory* of adaptive immunity; the immune process begins with a seminal perception of the healthy body. The reference set of antigen receptors are tuned to the state of wellness; disease is manifested by a significant fall, however slight, from a healthy pattern.

Obviously, this wellness view is at odds with the disease-oriented view developed by Western biomedicine as a corollary to the *germ-theory of disease*: According to the standard paradigm, health is a given; health is freedom from pathogenic agents such as bacteria, viruses, or malignant cells (44). The discovery of the DNA genetic code has added mutant or abnormal DNA to the causes of disease. Immune machine learning would suggest that immune wellness is not merely the absence of a specific disease but a particular body state, one that must be learned during early immune repertoire development. This shifts our perception of the immune response away from an exclusive preoccupation with disease and adds to the immune system the task of maintaining one's state of health (3). Wellness theory would suggest that a chronic or recurrent disease might arise from replacement of a healthy reference set of immune body data with an aberrant reference set; indeed, the chronic autoimmune disease lupus appears to be characterized by an aberrant autoantibody signature that is relatively stable (45)—the sick immune system views a lupus immune profile as if it were the patient's normal state. If this is true, then treatment of an autoimmune disease might aim at immune re-education toward a healthy reference profile rather than primarily at suppression of the autoreactivity. Likewise, successful allograft transplantation might be advanced by educating the host immune system to include key allo-antigens in the host's reference repertoire of health—this might explain the effect of allogeneic bone marrow transplantation of the induction of tolerance to an allogenic graft. Effective tumor immunotherapy, as we have mentioned in passing, deprives the tumor of its resemblance to healthy wellness—rejection then follows (27).

## Experimental Testing

Hypothesis and theory contribute to empirical science in two important ways: First, they can help initiate new thinking regarding known observations, and second, and most importantly, they can inspire new experiments. We have raised two related points that invite novel experimentation: the concept of a *Wellness Profile* and its function as a training set of data that guides the type of inflammatory immune response to variable test data.

The Wellness Profile hypothesis proposes that healthy individual humans (and by extension other mammals) share common sets of autoantibodies and TCR repertoires. This hypothesis was inspired by our finding that the cord bloods of different newborns are highly correlated in their IgM autoantibodies produced *in utero*. Healthy adults go on to modify their initial cord blood repertoires of IgM and IgG through physiological immune experience.

If indeed there is a Wellness Profile in adult life, then we predict that we will be able to discover a list of autoantibody reactivities shared by most healthy people. Some antibodies in this Wellness autoantibody list will be absent in people with chronic autoimmune disease. Indeed, we predict that we will find a number autoantibodies that are shared by people suffering from different chronic autoimmune diseases—a type of Illness Profile. We plan to carry out these experiments using the antigen microarray device developed by one of us (45); informatic analyses of sufficient numbers of samples will test whether our prediction is borne out.

The Wellness Profile hypothesis also includes TCR repertoires, which are technically more difficult to study. Shared, public TCR receptors have already been published, and we predict that public TCR sequences will include repertoire features that are shared by healthy people and absent in the TCR repertoires of people suffering from chronic autoimmunity problems or tumors. We can carry out such a study by informatic analysis of published TCR data from healthy “controls” compared to samples from persons with chronic autoimmune conditions or cancer.

We here have proposed that the immune inflammation phenotype is influenced by training sets of autoantigen reactivities arising during healthy development. This idea can be tested by introducing, during development, otherwise immunogenic antigens such as allogeneic cells to induce specific lifelong “tolerance” to specific allografts in inbred mice. We would predict that modified training sets of autoreactive autoantibody and TCR repertoires would be detected in these mice and would persist throughout adult life; these modified training reactivities would be added to the standard, shared profile of wellness present in the mice.

These predictions can be tested using a model of alloantigen tolerance induced in mice before birth *in utero* or shortly after birth. The newborn mice exposed to allo-antigens during development will manifest modified Wellness Profiles that include specific allo-antibodies and modified TCR repertoires; the mice with modified profiles should accept H2-specific allografts, according to our proposed theory. Adoptive transfer of modified TCR and autoantibody repertoires in inbred mice would make it possible to isolate the key elements in the transferred repertoires.

In contrast to inducing tolerance to foreign transplantation antigens, it appears that enhanced autoimmune T-cell mediated inflammation in adults can be induced in newborn mice by injection of selected autoimmune T cells: adult rats of the Fischer strain can mount T-cell proliferative responses to myelin basic protein but they resist developing inflammation that causes experimental autoimmune encephalomyelitis (EAE); however, injecting newborn Fisher rats with anti-MBP T cells renders the rats susceptible to inflammatory EAE induced by active immunization later in adult life (46); the injected T cells did not cause EAE in the newborn rats, but the injected T cells migrated to the thymus and spleen and persisted there. These early findings suggest that it might indeed be feasible to modulate a later inflammatory immune response by manipulating the developing T-cell repertoire. Some of the novel concepts outlined here do stimulate novel research programs.

## Coda

To summarize, the standard clonal selection paradigm fails to account for new findings that confound simple binary, self-non-self explanations of complex immune behavior. Here, we propose immune system computation, swarm intelligence, and experience-based training repertoires as strategies for intelligent, self-organizing body maintenance, healing, and protection.

## Author Contributions

All authors listed have made a substantial, direct and intellectual contribution to the work, and approved it for publication.

### Conflict of Interest Statement

The authors declare that the research was conducted in the absence of any commercial or financial relationships that could be construed as a potential conflict of interest.
